# Artificial intelligence in cardiopulmonary resuscitation: a systematic review

**DOI:** 10.1016/j.bjane.2026.844795

**Published:** 2026-07-12

**Authors:** Diogenes de Oliveira Silva, Giorgio Pretto, Saul Dominici, Antonio Carlos Aguiar Brandão, Plínio da Cunha Leal

**Affiliations:** a Anestech Information Technology Ltd., Florianópolis, SC, Brazil; b Universidade Federal do Maranhão, Department of Medicine, São Luís, MA, Brazil; c Universidade Federal de São Paulo, Postgraduate Program in Interdisciplinary Surgical Science, São Paulo, SP, Brazil; d Universidade do Vale do Sapucaí, Department of Anesthesiology, Pouso Alegre, MG, Brazil

**Keywords:** Artificial intelligence, Cardiopulmonary resuscitation, Clinical decision support systems, Emergency medical services, Heart arrest, Machine learning

## Abstract

Cardiac arrest remains a major cause of mortality and neurological disability, and its management depends on rapid recognition, effective resuscitation, and accurate post-arrest prognostication. Artificial intelligence (AI) has been increasingly investigated as a tool to support these stages of care. This narrative review summarizes recent evidence on AI applications in cardiac arrest, including prediction, recognition, intra-arrest support, prognostication, and cardiovascular risk stratification. Searches were conducted in PubMed/MEDLINE, Google Scholar, EMBASE, Scopus, IEEE Xplore, and ScienceDirect for studies published from January 2000 to June 2025. Studies addressing AI, machine learning, or deep learning in cardiac arrest-related contexts were descriptively synthesized according to clinical application, AI methodology, and care setting. Overall, 59 studies were included. Six application categories were identified: early prediction of cardiac arrest or clinical deterioration (n = 16), cardiac arrest recognition and emergency response activation (n = 3), intra-arrest CPR and defibrillation support (n = 6), post-cardiac arrest prognostication (n = 20), risk stratification for sudden cardiac death and malignant arrhythmias (n = 11), and related cardiovascular or perioperative prognostic models (n = 3). Deep learning and neural-network-based approaches were the most frequent methodologies (n = 35), followed by traditional machine learning (n = 18) and natural language processing (n = 6). AI showed particular promise for ECG-based prediction, temporal clinical trajectories, emergency call interpretation, waveform and defibrillation analyses, and neurological or mortality prognostication after cardiac arrest. However, the evidence remains heterogeneous, with many studies relying on retrospective datasets, selected populations, and limited external or prospective validation.

## Introduction

Cardiac Arrest (CA) constitutes a critical medical emergency characterized by an abrupt cessation of circulatory and respiratory function, resulting in interruption of oxygen supply to vital organs.^1^ This condition represents one of the leading causes of mortality, with an estimated incidence of approximately 8.27 cases per 1,000 hospitalizations.^2^ Despite significant advances in emergency medicine and resuscitation techniques over the past decades, survival rates remain challenging, with only 25% of patients surviving to hospital discharge and only 22% of survivors achieving a good neurological status.^3^

The management of cardiac arrest faces clinical challenges that directly impact patient outcomes, as there is a limited critical time window during which Cardiopulmonary Resuscitation (CPR) can be adequate, with the probability of success decreasing drastically with each minute of delay in recognition and initiation of resuscitation maneuvers.^4^ Late recognition of cardiac arrest and delayed CPR initiation are factors frequently associated with unfavorable outcomes. Additionally, cardiac arrest survivors frequently develop significant neurological complications as part of an unpredictable long-term prognosis.^5^ The complexity of cardiac arrest care is amplified by the need for rapid and precise decision-making in high-pressure environments, where healthcare professionals must integrate multiple clinical variables, interpret vital signs in real-time, and execute coordinated team interventions. Traditional prediction and risk assessment methods are based on clinical scores and guidelines that, although validated, do not exceed 50% accuracy in identifying high-risk patients.^6^

In this context, Artificial Intelligence (AI) offers opportunities to address limitations in cardiac arrest management. AI offers opportunities to address the limitations of conventional methods by processing large data volumes, identifying complex patterns that the human eye cannot detect, and providing real-time predictive analyses based on Electrocardiogram (ECG) data or multiple electronic health records.^7^ Beyond prediction, AI can optimize resuscitation quality through real-time feedback systems for the team and assistance in coordinating actions.^8^ Additionally, natural language processing systems can improve recognition of cardiac arrest in emergency calls, reducing the time between the event and the arrival of specialized assistance.^9^

The objective of this review was to provide an overview of recent advances, main methodological approaches, and potential clinical applications of AI in the prediction, detection, recognition, and management of cardiac arrest.

## Methods

This study constitutes a narrative review of the contemporary scientific literature on the application of Artificial Intelligence (AI) in cardiac arrest, with searches conducted across databases including PubMed/MEDLINE, Google Scholar, EMBASE (Excerpta Medica Database), Scopus, IEEE Xplore, and ScienceDirect.

The literature search spanned the period from January 2000 to June 2025 and was conducted between January and June 2025, employing combinations of keywords related to artificial intelligence and cardiac arrest, such as “artificial intelligence cardiac arrest”, “machine learning cardiopulmonary resuscitation”, “AI emergency medicine cardiac arrest”, “deep learning ECG cardiac arrest prediction”, “artificial intelligence CPR quality”, and “machine learning emergency medical services”, along with variations, synonyms, and Boolean combinations incorporating terms like “prediction”, “detection”, “recognition”, “Electrocardiography” (ECG/EKG), “emergency medicine”, “resuscitation”, “sudden cardiac death”, and “sudden cardiac arrest” to capture relevant publications.

Titles and abstracts of retrieved publications were screened for relevance, focusing on studies addressing AI, machine learning, or deep learning applications in cardiac arrest contexts, including prediction, detection, recognition, or optimization of Cardiopulmonary Resuscitation (CPR); preference was given to original research articles in peer-reviewed journals written in English or Portuguese, with emphasis on publications on recent technological, while excluding editorials, commentaries, conference abstracts lacking full data, and unrelated studies.

When available in the studies, algorithm performance metrics such as sensitivity, specificity, Area Under the Receiver Operating Characteristic Curve (AUROC), and F1-score were extracted, with studies descriptively categorized by primary application (cardiac arrest prediction, recognition, CPR optimization, or post-arrest outcome prediction), AI methodology employed, and clinical setting (e.g., in-hospital or out-of-hospital cardiac arrest, emergency departments, or intensive care units).

Due to heterogeneity in study designs, datasets, and evaluation metrics, findings were synthesized qualitatively through narrative analysis rather than quantitative meta-analysis.

Before final manuscript submission, all included references were checked for retraction notices and formal expressions of concern using PubMed, Crossref/Crossmark, and the Retraction Watch Database. No retracted articles or articles subject to formal expressions of concern were identified among the references included in the final manuscript.

## Results

The 59 included studies were grouped into six application categories according to their primary clinical purpose in cardiac arrest care: early prediction of cardiac arrest or clinical deterioration, cardiac arrest recognition and emergency response activation, intra-arrest support for CPR and defibrillation, post-cardiac arrest prognostication, risk stratification for sudden cardiac death and malignant arrhythmias, and related cardiovascular or perioperative prognostic models (Table 1).

**Table 1:** Application categories.

Application category	N	Articles	Description
Early prediction of cardiac arrest/clinical deterioration	16	Arnold et al.;^10^ Cho et al.;^11^ Fernandes et al.;^12^ Hu et al.;^13^ Jang et al.;^14^ Javan et al.;^15^ Kennedy et al.;^16^ Kim et al.;^17^ Kwon et al.;^18^ Kwon et al.;^19^ Lee et al.;^20^ Lee et al.;^21^ Lu et al.;^22^ Matam et al.;^23^ Ong et al.;^24^ Shamout et al.^25^	AI models used to predict cardiac arrest or clinical deterioration before the event, based on ECG, vital signs, laboratory data, clinical history, or patient trajectories.
Cardiac arrest recognition and emergency response activation	3	Blomberg et al.;^26^ Blomberg et al.;^27^ Chen et al.^28^	AI tools for recognizing cardiac arrest in emergency calls or improving emergency response logistics, including dispatcher support and AED.
Intra-arrest, CPR, and defibrillation support	6	He et al.;^29^ Howe et al.;^30^ Islam et al.;^31^ Liu et al.;^32^ Rad et al.;^33^ Yang et al.^34^	Studies applying AI during active resuscitation, including rhythm classification, waveform analysis, defibrillation outcome prediction, ROSC prediction, and biomedical signal processing during CPR.
Post-cardiac arrest prognostication	20	Al-Dury et al.;^35^ Amorim et al.;^36^ Andersson et al.;^37^ Chung et al.;^38^ Coult et al.;^39^ Elmer et al.;^40^ Ghassemi et al.;^41^ Harford et al.;^42^ Hirano et al.;^43^ Johnsson et al.;^44^ Jonas et al.;^45^ Kwon et al.;^46^ Le Duff et al.;^47^ Nanayakkara et al.;^48^ Park et al.;^49^ Pugin et al.;^50^ Seki et al.;^51^ Tjepkema-Cloostermans et al.;^52^ Tjepkema-Cloostermans et al.;^53^ Wagner et al.^54^	AI models for predicting survival, neurological outcome, mortality, recovery, or post-anoxic brain injury phenotypes after cardiac arrest.
Risk stratification for sudden cardiac death and malignant arrhythmias	11	Alonso et al.;^55^ Au-Yeung et al.;^56^ Ebrahimzadeh et al.;^57^ Lai et al.;^58^ Liu et al.;^59^ Liu et al.;^60^ Martinez-Alanis et al.;^61^ Moon et al.;^62^ Tylman et al.;^63^ Wu et al.;^64^ Zoni-Berisso et al.^65^	Studies estimating the risk of sudden cardiac death, malignant ventricular arrhythmias, tachyarrhythmias, NSTEMI, or major adverse cardiovascular events before cardiac arrest occurs.
Related cardiovascular or perioperative prognostic models	3	Chong et al.;^66^ Turton et al.;^67^ Wise et al.^68^	Peripheral cardiovascular or perioperative prognostic models with indirect relevance to the review, but not directly focused on cardiac arrest or CPR.

AED, Automated External Defibrillator; AI, Artificial Intelligence; CPR, Cardiopulmonary Resuscitation; ECG, Electrocardiogram; NSTEMI, Non-ST-elevation Myocardial Infarction; ROSC, Return of Spontaneous Circulation.

## Early prediction of cardiac arrest / clinical deterioration (16 studies)

Early prediction of cardiac arrest/clinical deterioration was the second largest category, comprising 16 studies.^10^, ^11^, ^12^, ^13^, ^14^, ^15^, ^16^, ^17^, ^18^, ^19^, ^20^, ^21^, ^22^, ^23^, ^24^, ^25^ These studies used AI models to anticipate cardiac arrest or clinical deterioration before the event, based on ECG data, vital signs, laboratory parameters, clinical history, or patient trajectories. This group included both ECG-based models and structured-data approaches. Kwon et al.^18^ developed a deep learning algorithm using 12-lead ECGs, achieving an AUROC of 0.913 in internal validation and 0.948 in external validation. More recently, Lu et al.^22^ introduced EIANet (ECG-Image-Aware Network), an ECG-image-aware deep learning model for emergency department cardiac arrest prediction, with an internal AUROC of 0.896 and external AUROC of 0.803.

These findings suggest that AI models may identify subtle physiological or electrocardiographic patterns associated with deterioration before cardiac arrest becomes clinically evident.

## Cardiac arrest recognition and emergency response activation (3 studies)

Cardiac arrest recognition and emergency response activation included three studies.^26^, ^27^, ^28^ These studies focused mainly on recognizing cardiac arrest in emergency calls or improving emergency response logistics, including dispatcher support and AED. Natural language processing and related AI-based approaches were used to support earlier recognition of out-of-hospital cardiac arrest and reduce delays in emergency response activation.

## Intra-arrest, CPR, and defibrillation support (6 studies)

Intra-arrest, CPR, and defibrillation support comprised six studies.^29^, ^30^, ^31^, ^32^, ^33^, ^34^ This category included AI applications during active resuscitation, such as rhythm classification, ECG waveform analysis, defibrillation outcome prediction, ROSC (Return of spontaneous circulation) prediction, and biomedical signal processing during CPR. These studies indicate that AI may support real-time decision-making during resuscitation by helping interpret complex physiological signals and potentially improving CPR and defibrillation strategies.

## Post-cardiac arrest prognostication (20 studies)

Post-cardiac arrest prognostication was the largest category, comprising 20 studies.^35^, ^36^, ^37^, ^38^, ^39^, ^40^, ^41^, ^42^, ^43^, ^44^, ^45^, ^46^, ^47^, ^48^, ^49^, ^50^, ^51^, ^52^, ^53^, ^54^ These studies evaluated AI models designed to predict survival, neurological outcomes, mortality, recovery patterns, or post-anoxic brain injury phenotypes after cardiac arrest. The predominance of this category indicates a strong research focus on post-resuscitation risk assessment and prognostic support, particularly for neurological recovery and mortality prediction.

## Risk stratification for sudden cardiac death and malignant arrhythmias (11 studies)

Risk stratification for sudden cardiac death and malignant arrhythmias included 11 studies.^55^, ^56^, ^57^, ^58^, ^59^, ^60^, ^61^, ^62^, ^63^, ^64^, ^65^ These studies estimated the risk of sudden cardiac death, malignant ventricular arrhythmias, tachyarrhythmias, NSTEMI (Non-ST-elevation myocardial infarction), or major adverse cardiovascular events before cardiac arrest occurs. Lai et al.^58^ developed the MAARS (Multimodal AI for Ventricular Arrhythmia Risk Stratification) model, which used cardiac magnetic resonance imaging to predict arrhythmic death in hypertrophic cardiomyopathy, achieving an overall accuracy of 89% and 93% accuracy among patients aged 40-60 years. This category broadens the scope of AI applications from immediate cardiac arrest prediction to preventive risk stratification in populations at increased cardiovascular risk.

## Cardiovascular or perioperative prognostic models

Related cardiovascular or perioperative prognostic models represented the smallest category, with three studies.^66^, ^67^, ^68^ These studies were not directly focused on cardiac arrest or CPR but were included because of their indirect relevance to cardiovascular or perioperative prognostication. Their inclusion highlights adjacent areas in which AI-based risk prediction may inform broader clinical decision-making related to acute cardiovascular deterioration.

## Analysis by AI methodology

### Deep Learning (35 studies)

Deep learning and neural-network-based approaches were the most frequent methodological strategies. These models included convolutional neural networks for ECG image analysis and biomedical signal processing, recurrent neural networks and long short-term memory networks for temporal trajectories, and artificial neural network models for clinical prediction or prognostic classification. They were mainly applied to early prediction of cardiac arrest or clinical deterioration, post-cardiac arrest prognostication, and risk stratification for sudden cardiac death or malignant arrhythmias.^13^^,^^14^^,^^17^, ^18^, ^19^, ^20^, ^21^, ^22^^,^^25^^,^^29^^,^^33^^,^^34^^,^^36^, ^37^, ^38^^,^^41^^,^^45^^,^^46^^,^^52^^,^^53^^,^^57^^,^^58^^,^^60^^,^^65^, ^66^, ^67^

### Traditional machine learning (18 studies)

Traditional machine learning methods, including support vector machines, random forests, Bayesian networks, supervised learning, data mining approaches, and machine learning scores, were identified across studies using structured clinical data, HRV-derived variables, waveform metrics, neuroimaging features, and cardiovascular risk predictors. These approaches were particularly frequent in intra-arrest defibrillation or ROSC prediction, post-arrest outcome prediction, and risk stratification for sudden cardiac death or major cardiovascular events.^10^^,^^11^^,^^15^^,^^16^^,^^23^^,^^24^^,^^30^, ^31^, ^32^^,^^35^^,^^39^^,^^40^^,^^42^, ^43^, ^44^^,^^47^, ^48^, ^49^, ^50^, ^51^^,^^54^, ^55^, ^56^^,^^59^^,^^61^^,^^63^^,^^64^^,^^68^

### Natural language processing (6 studies)

Natural language processing was used in studies involving emergency call analysis, textual clinical data, or automated extraction of risk factors from medical records. These applications were mainly related to cardiac arrest recognition, emergency response activation, and integration of unstructured text with clinical prediction models.^12^^,^^26^, ^27^, ^28^^,^^62^

Overall, deep learning models appeared more frequently in ECG-based prediction, temporal physiological data, and complex signal or image interpretation, whereas traditional machine learning approaches were more common in structured clinical prediction, waveform-based defibrillation analyses, and cardiovascular risk stratification. Natural language processing represented a smaller but clinically relevant group, especially for emergency call interpretation and extraction of information from unstructured records.

## Discussion

This narrative review summarizes current evidence on the use of artificial intelligence in cardiac arrest prediction, recognition, resuscitation support, and post-arrest prognostication. Overall, the included studies suggest that AI-based approaches have potential clinical relevance, particularly in ECG-based prediction and early detection of clinical deterioration.

Given the high-stakes nature of cardiac arrest care, AI applications in this field require particularly rigorous evaluation. Models intended to support prediction, recognition, resuscitation, or prognostication must demonstrate not only technical performance but also clinical validity, safety, interpretability, and reproducibility across different settings. The intersection between AI and emergency medicine also demands multidisciplinary expertise, as reliable development and implementation depend on both robust computational methods and a clear understanding of the clinical context in which these tools may be used.

The temporal analysis of studies reveals a clear progress in the technological sophistication of AI applications. The earliest studies (2018‒2019) focused mainly on traditional machine learning algorithms applied to structured clinical data. Starting in 2020, a transition toward more complex deep learning models has been observed, particularly in ECG analysis and biomedical signal processing.

The development of the EIANet model by Lu et al.^22^ represents a milestone in technological evolution, demonstrating how deep learning architectures can be specifically adapted for the clinical context. The incorporation of spatial attention modules and a customized loss function (Binary Recall Loss) illustrates the field's growing methodological sophistication.

The advantage of AI-based ECG analysis deserves special emphasis. The fact that algorithms can identify patterns in the QRS complex and ST segment that are imperceptible to the human eye suggests that significant predictive information is being underutilized in current clinical practice. This discovery has potential clinical relevance for the development of continuous monitoring and early warning systems.

External validation was clearly reported in only a small subset of the included studies. Kwon et al.^19^ externally validated an ECG-based deep learning algorithm in an independent hospital dataset, Lee et al.^20^ performed a multicentre validation of the deep learning-based early warning score, Lu et al.^22^ externally validated the EIANet model using an independent emergency department ECG-image dataset, and Tjepkema-Cloostermans et al.^53^ externally validated a deep learning model for postanoxic coma using data from additional hospitals. Most remaining studies relied on internal validation, cross-validation, holdout test sets, or registry-based train-test splits, which may overestimate real-world generalizability.

The study by Kwon et al.^19^ exemplifies both the potential and the challenges of external validation. Although the model demonstrated an AUROC of 0.948 in external validation, this performance was achieved in a hospital with characteristics similar to those of the development hospital. True generalization requires validation in more diverse populations and varied clinical contexts.

Studies with external validation have demonstrated greater robustness and generalization, though they often show slightly lower performance than internal validation, highlighting the importance of validation in independent populations. This is an implicit limitation of AI models, as they are trained on specific databases, and performance declines when applied to population data outside those databases.

The question of interpretability emerges as a critical challenge, particularly for deep learning models. Although these models demonstrate higher performance, their “black box” nature may limit clinical acceptance. The development of explainability techniques, such as attention maps and feature analysis, represents important progress but remains insufficient for many critical clinical applications.

Practical implementation of AI systems in real clinical environments faces significant challenges beyond technical performance. Issues of interoperability with hospital information systems, processing latency, and integration with existing clinical protocols are frequently underestimated in development studies.

Clinical confidence in AI systems requires not only high performance but also a thorough understanding of the decision-making mechanisms. The fact that the EIANet model identifies the ST segment as a region of interest provides important clinical validation, since ST segment alterations are known to be associated with adverse cardiac events.

The EIANet model, which uses ECG images obtained during screening, represents a pragmatic approach that recognizes the realities of clinical workflows. This strategy may facilitate the practical implementation compared to approaches that require significant modifications to existing processes. Results suggest AI applications may influence multiple aspects of cardiac arrest care. Early prediction capacity may enable preventive interventions that avoid cardiac arrest occurrence or improve medical team preparedness.

The MAARS model developed by Lai et al.^58^ exemplifies the potential of AI for personalized care. These models may support future risk stratification strategies, but their role in routine care remains limited by the need for broader external validation, interpretability, workflow integration, and evaluation in prospective clinical settings.

The successful implementation of AI systems for cardiac arrest requires careful consideration of multiple factors. First, there is a need for adequate training of healthcare professionals to interpret and act based on AI predictions. Second, the development of clinical protocols that safely and effectively incorporate AI recommendations.

Experience with traditional early warning systems suggests that mere availability of predictive information does not guarantee improvement in clinical outcomes. Effectiveness depends on the healthcare system's capacity to appropriately respond to generated alerts.

## Future perspectives

The future evolution of AI in cardiac arrest will likely follow several promising directions. The development of multimodal models that integrate ECG, vital signs, laboratory data, and medical images may offer promising performance in selected datasets compared to current unimodal models.

The application of federated learning techniques can address privacy and generalization issues, allowing the development of robust models without direct sharing of sensitive data between institutions. This approach is particularly relevant for cardiac arrest, where collaboration between multiple institutions is essential to obtain sufficiently large datasets.

This review identifies several important gaps that require future research, as there is an urgent need for randomized controlled trials to evaluate the real impact of AI implementation on clinical outcomes. Most current studies focus on the technical performance of algorithms, but evidence of real clinical benefits remains limited.

Furthermore, cost-effectiveness studies are necessary to inform implementation decisions in healthcare systems with limited resources. High technical performance must be balanced against the costs of implementation and maintenance.

Research on acceptance and usability by healthcare professionals is essential to ensure successful adoption. Resistance to change and concerns about the substitution of clinical judgment may limit the implementation of even technically superior systems.

## Ethical and regulatory considerations

The application of AI in cardiac arrest raises important ethical questions that require careful consideration. Automation of decisions that may impact the life or death of patients requires rigorous ethical standards and adequate human supervision.

The issue of responsibility in the event of system failures is particularly complex. When an AI algorithm fails to predict a cardiac arrest that subsequently occurs, or when it generates a false positive leading to unnecessary interventions, attribution of responsibility among developers, implementers, and clinical users remains undefined.

The regulation of AI systems in medicine is evolving rapidly, but it still faces significant challenges. The dynamic nature of machine learning algorithms, which may continue learning after implementation, challenges traditional regulatory frameworks based on static medical devices. The development of standards for validating, continuously monitoring, and updating AI systems in medicine is essential to ensure long-term safety and efficacy.

## Limitations

This review has several limitations that should be acknowledged. First, the methodological heterogeneity of included studies allows only a narrative review. Second, the rapid evolution of the field means that important new studies may have been published during the conduct of this review, potentially altering the conclusions.

Finally, most included studies originate from developed countries with advanced technological infrastructures, which limits the generalizability of the findings to contexts with limited resources.

## Conclusion

This narrative review indicates that AI has potential applications in cardiac arrest, including prediction, recognition of clinical deterioration, support for CPR quality, and post-arrest prognostication. Several studies reported promising algorithmic performance, particularly for ECG-based and structured-data models. However, the current evidence is heterogeneous, and most findings are based on retrospective datasets, selected populations, or limited validation settings.

Despite encouraging results, the clinical implementation of AI in cardiac arrest remains at an early stage. Limited external validation, scarce prospective evaluation, insufficient real-world implementation data, and uncertainty regarding workflow integration, interpretability, and clinical response to AI-generated alerts remain major barriers. Future research should prioritize multicenter external validation, prospective clinical studies, randomized or pragmatic implementation trials, cost-effectiveness analyses, and usability assessments involving healthcare professionals. Until such evidence is available, AI should be considered a promising adjunctive tool rather than a replacement for clinical judgment or established resuscitation protocols.

## Data availability statement

No new data were created or analyzed in this study. Data sharing is not applicable to this article.

## AI assistance disclosure

AI-assisted tools were used to support language editing, textual organization, formatting, and refinement of the manuscript, including improvement of clarity, grammar, consistency, and reference presentation. These tools were not used to generate original data, perform independent study selection, conduct statistical analyses, or make clinical or scientific interpretations. All AI-assisted suggestions were critically reviewed, verified, and edited by the authors, who take full responsibility for the accuracy, integrity, and final content of the manuscript.

## Conflicts of interest

The authors declare no conflicts of interest for this review.

## References

[1] Viderman D., Abdildin Y.G., Batkuldinova K., Badenes R., Bilotta F. (2023). Artificial intelligence in resuscitation: a scoping review. J Clin Med.

[2] Morrison L.J., Neumar R.W., Zimmerman J.L. (2013). Strategies for improving survival after in-hospital cardiac arrest in the United States: 2013 consensus recommendations: a consensus statement from the American Heart Association. Circulation.

[3] Girotra S., Nallamothu B.K., Spertus J.A. (2012). Trends in survival after in-hospital cardiac arrest. N Engl J Med.

[4] Merchant R.M., Yang L., Becker L.B. (2011). Incidence of treated cardiac arrest in hospitalized patients in the United States. Crit Care Med.

[5] Nolan J.P., Berg R.A., Andersen L.W. (2019). Cardiac arrest and cardiopulmonary resuscitation outcome reports: update of the Utstein Resuscitation Registry template for in-hospital cardiac arrest: a consensus report from a Task Force of the International Liaison Committee on Resuscitation. Circulation.

[6] Churpek M.M., Yuen T.C., Edelson DP. (2013). Risk stratification of hospitalized patients on the wards. Chest.

[7] Rasool A., Tao R., Kashif K., Khan W., Agbedanu P., Choudhry N. (2020). Proceedings of the 202012th International Conference on Machine Learning and Computing.

[8] Toy J., Bosson N., Schlesinger S., Gausche-Hill M., Stratton S. (2023). Artificial intelligence to support out-of-hospital cardiac arrest care: a scoping review. Resusc Plus.

[9] Zace D., Semeraro F., Schnaubelt S. (2025). Artificial intelligence in resuscitation: a scoping review. Resusc Plus.

[10] Arnold J., Davis A., Fischhoff B. (2019). Comparing the predictive ability of a commercial artificial intelligence early warning system with physician judgement for clinical deterioration in hospitalised general internal medicine patients: a prospective observational study. BMJ Open.

[11] Cho K.J., Kwon O., Kwon J.M. (2020). Detecting patient deterioration using artificial intelligence in a rapid response system. Crit Care Med.

[12] Fernandes M., Mendes R., Vieira S.M. (2020). Risk of mortality and cardiopulmonary arrest in critical patients presenting to the emergency department using machine learning and natural language processing. PLoS One.

[13] Hu S.B., Wong D.J.L., Correa A., Li N., Deng JC. (2016). Prediction of clinical deterioration in hospitalized adult patients with hematologic malignancies using a neural network model. PLoS One.

[14] Jang D.H., Kim J., Jo Y.H. (2020). Developing neural network models for early detection of cardiac arrest in emergency department. Am J Emerg Med.

[15] Javan S.L., Sepehri M.M., Javan M.L., Khatibi T. (2019). An intelligent warning model for early prediction of cardiac arrest in sepsis patients. Comput Methods Programs Biomed.

[16] Kennedy C.E., Aoki N., Mariscalco M., Turley JP. (2015). Using time series analysis to predict cardiac arrest in a PICU. Pediatr Crit Care Med.

[17] Kim J., Chae M., Chang H.J., Kim Y.A., Park E. (2019). Predicting cardiac arrest and respiratory failure using feasible artificial intelligence with simple trajectories of patient data. J Clin Med.

[18] Kwon J., Lee Y., Lee Y., Lee S., Park J. (2018). An algorithm based on deep learning for predicting in-hospital cardiac arrest. J Am Heart Assoc.

[19] Kwon J.M., Kim K.H., Jeon K.H., Lee S.Y., Park J., Oh B.H. (2020). Artificial intelligence algorithm for predicting cardiac arrest using electrocardiography. Scand J Trauma Resusc Emerg Med.

[20] Lee Y., Kwon J.M., Lee Y., Park H., Cho H., Park J. (2018). Deep learning in the medical domain: predicting cardiac arrest using deep learning. Acute Crit Care.

[21] Lee Y.J., Cho K.J., Kwon O. (2021). A multicentre validation study of the deep learning-based early warning score for predicting in-hospital cardiac arrest in patients admitted to general wards. Resuscitation.

[22] Lu S.C., Chen G.Y., Liu A.S. (2025). Deep learning-based electrocardiogram model (EIANet) to predict emergency department cardiac arrest: development and external validation study. J Med Internet Res.

[23] Matam B.R., Duncan H., Lowe D. (2019). Machine learning based framework to predict cardiac arrests in a paediatric intensive care unit: prediction of cardiac arrests. J Clin Monit Comput.

[24] Ong M.E.H., Ng C.H.L., Goh K. (2012). Prediction of cardiac arrest in critically ill patients presenting to the emergency department using a machine learning score incorporating heart rate variability compared with the modified early warning score. Crit Care.

[25] Shamout F.E., Zhu T., Sharma P., Watkinson P.J., Clifton DA. (2020). Deep interpretable early warning system for the detection of clinical deterioration. IEEE J Biomed Health Inform.

[26] Blomberg S.N., Christensen H.C., Lippert F. (2021). Effect of machine learning on dispatcher recognition of out-of-hospital cardiac arrest during calls to emergency medical services. JAMA Netw Open.

[27] Blomberg S.N., Folke F., Ersbøll A.K. (2019). Machine learning as a supportive tool to recognize cardiac arrest in emergency calls. Resuscitation.

[28] Chen C.H., Hsieh J.G., Cheng S.L., Lin Y.L., Lin P.H., Jeng JH. (2020). Emergency department disposition prediction using a deep neural network with integrated clinical narratives and structured data. Int J Med Inform.

[29] He M., Lu Y., Zhang L., Zhang H., Gong Y., Li Y. (2016). Combining amplitude spectrum area with previous shock information using neural networks improves prediction performance of defibrillation outcome for subsequent shocks in out-of-hospital cardiac arrest patients. PLoS One.

[30] Howe A., Escalona O.J., Di Maio R. (2014). A support vector machine for predicting defibrillation outcomes from waveform metrics. Resuscitation.

[31] Islam S., Bentahar J., Cohen R., Rjoub G. (2025). A multimodal unsupervised machine learning approach for biomedical signal processing during cardiopulmonary resuscitation. Inf Sci.

[32] Liu N., Koh Z.X., Goh J. (2014). Prediction of adverse cardiac events in emergency department patients with chest pain using machine learning for variable selection. BMC Med Inform Decis Mak.

[33] Rad A.B., Eftestol T., Engan K. (2017). ECG-based classification of resuscitation cardiac rhythms for retrospective data analysis. IEEE Trans Biomed Eng.

[34] Yang Z., Yang Z., Lu W., Harrison R.G., Eftestøl T., Steen PA. (2005). A probabilistic neural network as the predictive classifier of out-of-hospital defibrillation outcomes. Resuscitation.

[35] Al-Dury N., Ravn-Fischer A., Hollenberg J. (2020). Identifying the relative importance of predictors of survival in out of hospital cardiac arrest: a machine learning study. Scand J Trauma Resusc Emerg Med.

[36] Amorim E., van der Stoel M., Nagaraj S.B. (2019). Quantitative EEG reactivity and machine learning for prognostication in hypoxic-ischemic brain injury. Clin Neurophysiol.

[37] Andersson P., Johnsson J., Björnsson O. (2021). Predicting neurological outcome after out-of-hospital cardiac arrest with cumulative information; development and internal validation of an artificial neural network algorithm. Crit Care.

[38] Chung C.C., Chiu W.T., Huang Y.H., Chan L., Hong C.T., Chiu H.W. (2021). Identifying prognostic factors and developing accurate outcome predictions for in-hospital cardiac arrest by using artificial neural networks. J Neurol Sci.

[39] Coult J., Blackwood J., Sherman L., TD Rea, Kudenchuk P.J., Kwok H. (2019). Ventricular fibrillation waveform analysis during chest compressions to predict survival from cardiac arrest. Circ Arrhythm Electrophysiol.

[40] Elmer J., Coppler P.J., May T.L. (2020). Unsupervised learning of early post-arrest brain injury phenotypes. Resuscitation.

[41] Ghassemi M.M., Amorim E., Alhanai T. (2019). Quantitative electroencephalogram trends predict recovery in hypoxic-ischemic encephalopathy. Crit Care Med.

[42] Harford S., Darabi H., Del Rios M. (2019). A machine learning based model for out of hospital cardiac arrest outcome classification and sensitivity analysis. Resuscitation.

[43] Hirano Y., Kondo Y., Sueyoshi K., Okamoto K., Tanaka H. (2021). Early outcome prediction for out-of-hospital cardiac arrest with initial shockable rhythm using machine learning models. Resuscitation.

[44] Johnsson J., Björnsson O., Andersson P. (2020). Artificial neural networks improve early outcome prediction and risk classification in out-of-hospital cardiac arrest patients admitted to intensive care. Crit Care.

[45] Jonas S., Rossetti A.O., Oddo M., Jenni S., Favaro P., Zubler F. (2019). EEG-based outcome prediction after cardiac arrest with convolutional neural networks: performance and visualization of discriminative features. Hum Brain Mapp.

[46] Kwon J.M., Jeon K.H., Kim H.M. (2019). Deep-learning based out-of-hospital cardiac arrest prognostic system to predict clinical outcomes. Resuscitation.

[47] Le Duff F., Muntean C., Cuggia M., Mabo P. (2004). Predicting survival causes after out of hospital cardiac arrest using data mining method. Stud Health Technol Inform.

[48] Nanayakkara S., Fogarty S., Tremeer M. (2018). Characterising risk of in-hospital mortality following cardiac arrest using machine learning: a retrospective international registry study. PLoS Med.

[49] Park J.H., Shin S.D., Song K.J. (2019). Prediction of good neurological recovery after out-of-hospital cardiac arrest: a machine learning analysis. Resuscitation.

[50] Pugin D., Hofmeister J., Gasche Y. (2020). Resting-state brain activity for early prediction outcome in postanoxic patients in a coma with indeterminate clinical prognosis. AJNR Am J Neuroradiol.

[51] Seki T., Tamura T., Suzuki M. (2019). SOS-KANTO 2012 Study Group. Outcome prediction of out-of-hospital cardiac arrest with presumed cardiac etiology using an advanced machine learning technique. Resuscitation.

[52] MC Tjepkema-Cloostermans, Hofmeijer J., Beishuizen A. (2017). Cerebral recovery index: reliable help for prediction of neurologic outcome after cardiac arrest. Crit Care Med.

[53] MC Tjepkema-Cloostermans, Lourenço C., Ruijter B. (2019). Outcome prediction in postanoxic coma with deep learning. Crit Care Med.

[54] Wagner F., Hänggi M., Weck A., Pastore-Wapp M., Wiest R., Kiefer C. (2020). Outcome prediction with resting-state functional connectivity after cardiac arrest. Sci Rep.

[55] Alonso D.H., Wernick M.N., Yang Y., Germano G., Berman D.S., Slomka P. (2019). Prediction of cardiac death after adenosine myocardial perfusion SPECT based on machine learning. J Nucl Cardiol.

[56] Au-Yeung W.T.M., Reinhall P.G., Bardy G.H., Brunton SL. (2018). Development and validation of warning system of ventricular tachyarrhythmia in patients with heart failure with heart rate variability data. PLoS One.

[57] Ebrahimzadeh E., Pooyan M., Bijar A. (2014). A novel approach to predict sudden cardiac death using nonlinear and time-frequency analyses from HRV signals. PLoS One.

[58] Lai C., Yin M., Kholmovski E.G. (2025). Multimodal AI to forecast arrhythmic death in hypertrophic cardiomyopathy. Nat Cardiovasc Res.

[59] Liu N., Ho A.F.W., Pek P.P. (2020). Prediction of ROSC after cardiac arrest using machine learning. Stud Health Technol Inform.

[60] Liu X., Liu T., Zhang Z. (2021). TOP-Net prediction model using bidirectional long short-term memory and medical-grade wearable multisensor system for tachycardia onset: algorithm development study. JMIR Med Inform.

[61] Martinez-Alanis M., Bojorges-Valdez E., Wessel N., Lerma C. (2020). Prediction of sudden cardiac death risk with a support vector machine based on heart rate variability and heartprint indices. Sensors (Basel).

[62] Moon S., Liu S., Scott C.G. (2019). Automated extraction of sudden cardiac death risk factors in hypertrophic cardiomyopathy patients by natural language processing. Int J Med Inform.

[63] Tylman W., Waszyrowski T., Napieralski A., Kamiński M., Trafidło T., Kulesza Z. (2016). Real-time prediction of acute cardiovascular events using hardware-implemented Bayesian networks. Comput Biol Med.

[64] Wu C.C., Hsu W.D., Islam M.M. (2019). An artificial intelligence approach to early predict non-ST-elevation myocardial infarction patients with chest pain. Comput Methods Programs Biomed.

[65] Zoni-Berisso M., Molini D., Viani S., Mela G.S., Delfino L. (2001). Noninvasive prediction of sudden death and sustained ventricular tachycardia after acute myocardial infarction using a neural network algorithm. Ital Heart J.

[66] Chong C.F., Li Y.C., Wang T.L., Chang H. (2003). Stratification of adverse outcomes by preoperative risk factors in coronary artery bypass graft patients: an artificial neural network prediction model. AMIA Annu Symp Proc.

[67] Turton E.P., Scott D.J., Delbridge M., Snowden S., Kester RC. (2000). Ruptured abdominal aortic aneurysm: a novel method of outcome prediction using neural network technology. Eur J Vasc Endovasc Surg.

[68] Wise E.S., Hocking K.M., Brophy CM. (2015). Prediction of in-hospital mortality after ruptured abdominal aortic aneurysm repair using an artificial neural network. J Vasc Surg.

